# Scaling up the sono-enzymatic coating of cotton textiles with antimicrobial silver-phenolated lignin nanocomposites

**DOI:** 10.1016/j.ultsonch.2025.107609

**Published:** 2025-10-09

**Authors:** Garima Rathee, Jeniffer Blair, Antonio Puertas-Segura, Kristina Ivanova, Guillem Ferreres Cabanes, Tzanko Tzanov

**Affiliations:** Grup de Biotecnologia Molecular i Industrial, Departament d’Enginyeria Química, Universitat Politècnica de Catalunya (UPC-BarcelonaTech), Rambla de Sant Nebridi 22, 08222 Terrassa (Barcelona), Spain

**Keywords:** Silver-phenolated lignin nanoparticles, Gallic acid, Laccase, Enzymatic grafting, Sonochemistry, Ultrasonic coating, Roll-to-roll processing, Medical cotton textiles, Hospital laundering, Biocompatibility, Silver release

## Abstract

The pressing demand for controlling infections in healthcare facilities has prompted the development of highly efficient antimicrobial textiles. Herein, we present a simple and scalable approach for engineering durable antimicrobial cotton textiles using a single-step sono-enzymatic process. The coating process involves simultaneous laccase enzyme-catalysed gallic acid (GA) oxidation under ultrasonic conditions (20 kHz frequency, 17.30 W/cm^2^ power intensity, 35 % amplitude, 50 °C, 30 min) using a Ti-horn ultrasonic transducer, generating a bio-based adhesive network that engages both GA and the oxidised phenolic shell of antimicrobial silver phenolated lignin nanoparticles (AgPLNPs), and their deposition on the fabrics. Three coating formulations were investigated: cotton fabric ultrasonicated with AgPLNPs alone (M1), with AgPLNPs and laccase (M2), and with AgPLNPs, GA, and laccase (M3). The AgPLNPs are firmly embedded on the cotton fabric, eliminating the need for fabric pre-activation or post-treatment. They are biocompatible, possess broad-spectrum antibacterial activity against Gram-positive (*Staphylococcus aureus*) and Gram-negative (*Pseudomonas aeruginosa* and *Escherichia coli*) bacteria, and have significantly less propensity to induce bacterial resistance than conventional antibiotics, showing only 2–4 fold increases in MIC compared to 128–2048 fold increases for ciprofloxacin and ampicillin. The resulting AgPLNPs-GA-coated textiles (M3) demonstrated durable antibacterial properties, retaining >95 % antibacterial efficacy after 60 hospital laundry cycles at 75 °C, following international thermal disinfection guidelines (98.6–99.8 % depending on bacterial strain). Additionally, the coated fabrics were biocompatible and hydrophobic (contact angle: 116.1 ± 1.5°), enhancing their benefits in medical environments where body fluid repellence is crucial for maintaining hygiene and preventing the spread of pathogens. The coating was successfully scaled up from laboratory samples (10 × 10 cm) to industrial-scale processing of textiles with dimensions 5 × 0.5 m using a continuous roll-to-roll sonochemical pilot. This novel coating approach shows promise for creating exceptionally durable antimicrobial and biocompatible fabrics for medical purposes.

## Highlights

• A green, one-step sono-enzymatic coating enables durable antimicrobial cotton textiles.
• Laccase-oxidised GA forms a crosslinked phenolic network with AgPLNPs ensuring long-lasting antimicrobial activity.
• The coated textiles retain antibacterial performance after 60 hospital laundry cycles.
• Broad-spectrum action against *S. aureus*, *P. aeruginosa*, and *E. coli* combined with demonstrated biocompatibility.
• Pilot-scale R2R sono-enzymatic coating confirms the industrial scalability of the process.

## Graphical abstract

**Figure:**
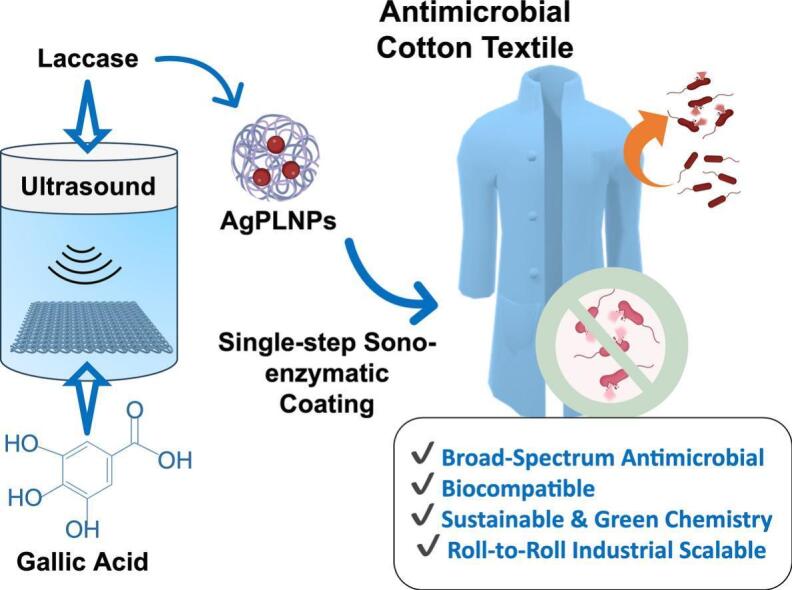

## 1 Introduction

Healthcare-associated infections (HAIs) pose a severe global threat, affecting 7 % of patients in high-income countries and 15 % in low/middle-income countries in acute care hospitals, with rates 2–3 times higher in intensive care units. With an estimated 8.9 million annual HAIs in the EU/EEA alone and mortality rates reaching 10 % among infected patients, these infections significantly contribute to increased healthcare costs, extended hospital stays, and patient mortality [1,2]. These numbers emphasise the urgent need for improved infection prevention strategies globally.

Multidrug-resistant bacteria, which emerged due to the misuse and overuse of antibiotics, have significantly decreased the treatment outcomes even with the latest resort antibiotics at clinically prescribed dosages. It is projected that by the year 2050, the mortality rate caused by bacterial infections will exceed 100 million [2]. A promising strategy to curb the spread of resistant bacterial strains is transforming the hospitals into pathogen-free, patient-safe environments by integrating antibacterial materials, such as textiles, that demonstrate both efficacy and durability under clinical conditions [3,4].

A wide range of antimicrobial agents has been used to this end, including organic compounds (quaternary ammonium compounds [5], triclosan [6], and N-halamines [7]), synthetic or natural polymers (chitosan [8], lignin [9], and antimicrobial natural dyes [10]), and metal-based materials (copper [11], silver [12], and zinc [13], and their oxides [4,14]). Despite the potential of these antimicrobial textile coatings, their efficacy and widespread adoption are hindered by poor coating durability (typically limited to 10–20 wash cycles), toxicity due to leaching of actives, and costly upscaling challenges [15]. Additionally, traditional methods such as padding [16], spray coating [17], and screen printing [18], among others, for fabricating functional textiles often require time-consuming and harsh fabric pre- and post-treatments to ensure coating stability and hence the durability of the antimicrobial effect.

Ultrasound (US) coating is a fast, cost-effective, and environmentally sustainable technology that provides ready-to-use surface-nanostructured products. This method was recognised by IUPAC as one of the top ten emerging coating technologies in 2021, underscoring the durability of the obtained antibacterial textiles [19]. US coating technology has also been adopted by industries, among which stand Nanosono [20], Sonovia Tech [21,22] and Sono-Tek [23], leveraging the potential of this innovative method for the production of advanced textiles with antibacterial, water-repellent, and flame-retardant finishes among others. The effectiveness of US arises from acoustic cavitation, in which collapsing bubbles in the liquid propel the adjacent nanoparticles (NPs) toward the fabric surface, forming robust coatings without damaging the fibres [24]. This technology has been used to synthesise and coat metal (e.g., Cu and Ag) [25,26] and metal oxide (e.g., CuO and ZnO) NPs [27,28], as well as to generate hybrid coatings on textiles combining chitosan and ZnO NPs to prevent bacterial colonisation [8]. Despite the antimicrobial effectiveness of the coating, the release of metal ions or NPs presents potential toxicity to humans and the environment [29,30]. This necessitates careful design approaches that minimise silver leaching while maintaining antimicrobial efficacy, particularly considering the environmental impact of washing effluents from repeated laundery cycles. Our group has used extensively the US to produce durable antimicrobial textiles [14,31]. In one study, we developed a method that combines sonochemical coating with *in-situ* generation of gallic acid (GA)-based adhesive upon cross-linking by the oxidative enzyme laccase, resulting in durable antimicrobial treatment [14]. The oxidative enzyme laccase enables the polymerisation of phenolic compounds and their grafting onto materials through hydrophobic interactions and hydrogen bonding [32,33]. Recent advances in antimicrobial textiles have explored various approaches, including metal–organic frameworks [34], surface functionalisation strategies [35], and nanocomposite coatings [36]. While our group has previously developed sono-enzymatic approaches using ZnO NPs [14] and antimicrobial AgPLNPs for wound dressings [37] urinary catheter coatings [38] and advanced water treatment [39], limitations remain in existing antimicrobial textile approaches regarding durability under repeated washing (typically 10–20 cycles), scalability to industrial production, and long-term biocompatibility. Most existing approaches require either harsh pre-treatment conditions, multi-step processes, or show significant activity loss after washing.

**Scheme 1:**
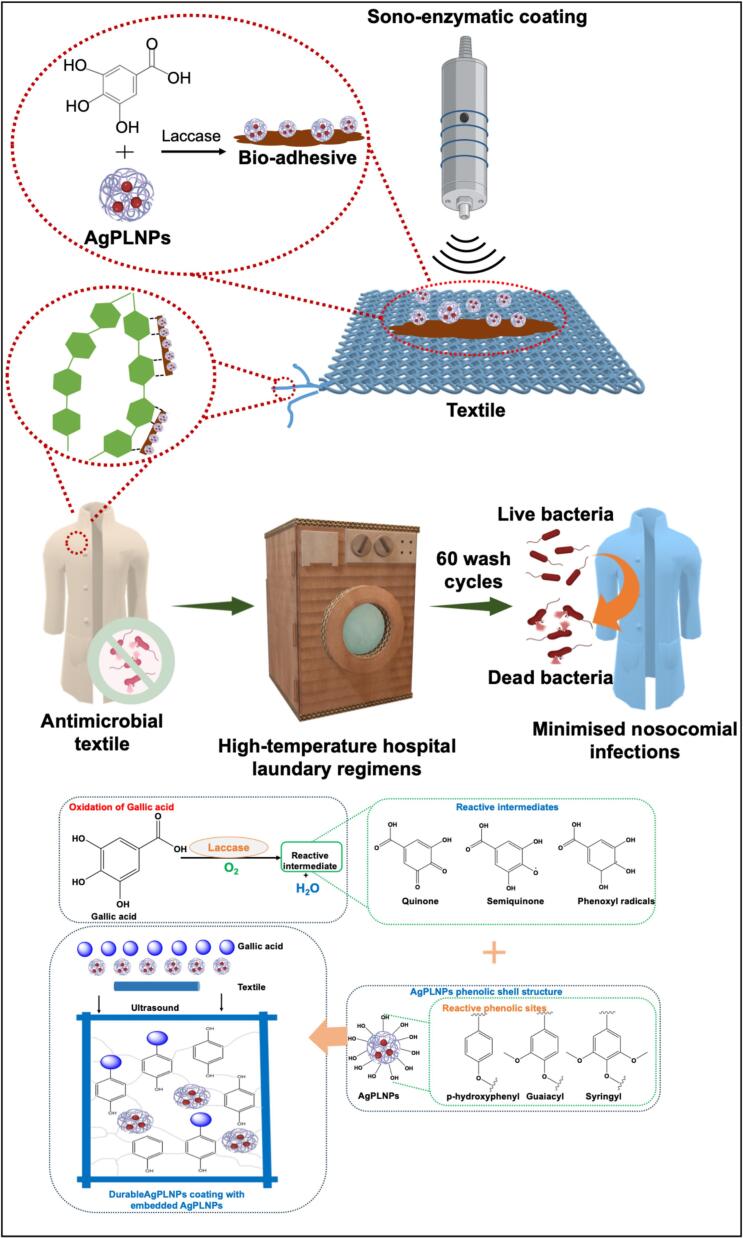
Schematic illustration of antimicrobial textile coating via single-step sono-enzymatic crosslinking of gallic acid and AgPLNPs. Laccase-catalysed oxidation generates reactive intermediates (quinones, semiquinones, phenoxy radicals) that undergo C-C and C-O-C coupling reactions with AgPLNPs phenolic shell components, forming a durable bio-adhesive coating with sustained antimicrobial efficacy through 60 washing cycles.

The present study overcomes these limitations by introducing the first single-step sono-enzymatic approach that integrates AgPLNPs with GA, enzymatically crosslinked and deposited onto textiles. This work advances beyond our previous publications through four key innovations: (i) the novel integration of AgPLNPs (previously used in wound dressings [37]) with specific enzymatic crosslinking chemistry to provide coating stability, (ii) the demonstration of unprecedented washing durability (>95 % efficacy after 60 cycles, compared to the typical limitation of 10–20 cycles, (iii) the first successful scale-up from laboratory scale (0.01 m^2^) to pilot-scale roll-to-roll (R2R) processing (2.5 m^2^), representing 250-fold scale increase, and (iv) systematic evaluation of resistance development potential, benchmarked against conventional antibiotics under identical conditions, for the simultaneous deposition of AgPLNPs embedded in a GA bioadhesive (Scheme 1). The coating process involves the simultaneous laccase-induced oxidation and sonochemical deposition of a bio-based, substrate-independent adhesive composed of crosslinked oxidised GA and phenolic-shell AgPLNPs onto the fabric. The phenolic shell enhances the biocompatibility of otherwise toxic silver NPs, whereas laccase-assisted crosslinking improves coating durability and modulates silver ion release, thereby extending the therapeutic effect while minimising toxicity [40]. The antibacterial activity was evaluated against healthcare-associated pathogens (S. aureus, P. aeruginosa, E. coli), with biocompatibility validated using human fibroblasts and keratinocytes. The process was successfully upscaled using a R2R coating pilot under the EU-project SONO The process was successfully upscaled using a roll-to-roll (R2R) coating pilot within the framework of the EU project SONO [41], with coating durability confirmed after 60 high-temperature hospital laundry cycles.

## 2 Methods

### 2.1 Materials

DAVO STAR IMPEX SRL, Romania, supplied the cotton fabric. PLT innovations from Switzerland provided the protobindTM 6000 lignin obtained from the agricultural feedstocks (specifications: sulfur-free (∼90 %), M_w_ ∼3500 g/mol, M_n_ ∼1000 g/mol, phenolic, aliphatic and acidic –OH contents (∼2.9, 2.6 and 0.8 mmol/g, respectively. ACROS Organics provided tannic acid, GA, and acetosyringone. Laccase from *Aspergillus* sp. (Sigma-Aldrich), acetic acid, sodium acetate, phosphate-buffered saline (PBS), Mueller-Hinton broth (MHB), cetrimide agar, Baird-Parker agar, and coliform agar were purchased from Sigma-Aldrich. Invitrogen provided the cell viability kit. New Enzymes (Portugal) provided the industrial laccase product NewLite Base 268. *S. aureus* (ATCC 25923), *P. aeruginosa* (ATCC 10145), *E. coli* (ATCC 25922), human keratinocyte (HaCaT) and fibroblast (ATCC-CRL-4001, BJ-5ta) cell lines were obtained from American Type Culture Collection (ATCC LGC Standards, Spain). Colorcenter (Spain) provided the non-ionic surfactant Cotemol NI.

### 2.2 Synthesis of AgPLNPs

AgPLNPs were produced following a previously reported protocol [38]. Initially, a 3: 2 lignin/Ag ratio (v/v) was prepared by adding AgNO_3_ solution (4 mg/mL) into phenolated lignin solution (10 mg/mL, pH 8) and then ultrasonicated using VCX 750 Ti-horn (20 kHz, 50 % amplitude, 60 °C) for 2 h. The synthesised AgPLNPs were first centrifuged at 20,000*g* for 20 min. After centrifugation, the AgPLNPs were washed twice with distilled water to remove soluble impurities. The suspension was centrifuged at 500*g* for 10 min to remove the unreacted lignin. Lastly, the AgPLNPs were lyophilised using Telstar LyoAlfa 15 lyophiliser and brown-coloured AgPLNP powder was obtained.

### 2.3 Characterisation of AgPLNPs

#### 2.3.1 Physicochemical characterisation

The hydrodynamic size, polydispersity index (PDI), and zeta potential of AgPLNPs was recorded on Zetasizer Nano Z (Malvern Instruments Inc., UK). The X-ray diffraction (XRD) spectrum was obtained using Bruker D8 Advance diffractometer (Bruker Inc., USA). The morphological data was collected using a FEI Tecnai G2 F20 high-resolution microscope equipped with a 200 kV field emission gun (FEI Inc., USA) and complimented with energy dispersive (EDX) spectroscopy. The molecular composition and functional groups of AgPLNPs were evaluated using attenuated total reflectance-Fourier transformed infrared spectroscopy (FTIR-ATR) (PerkinElmer/FTIR Spectrum 100R, USA). The phenolic content of the AgPLNPs was determined spectrophotometrically using the Folin–Ciocalteu method [37].

#### 2.3.2 Silver content

The silver content in the NPs and its reduction yield were determined using inductively coupled plasma mass spectrometry (ICP-MS). Briefly, 20 mg of lyophilised AgPLNPs were digested with 2 mL of 20 % nitric acid at 70 °C for 24 h. The digested samples were diluted with ultrapure water to obtain 2 % nitric acid solutions and filtered through 0.22 μm pore PES membranes to remove remaining solids. The ^107^Ag content was measured using an ICP-MS system (Model 7800, Agilent Technologies, Spain) to determine both the silver weight percentage in the dried nanoparticles and the percentage of initial Ag^+^ that was reduced to Ag^0^ and incorporated into the NPs. The results are presented as mean values ± standard deviations (n = 3).

#### 2.3.3 Antibacterial activity

The antibacterial performance of AgPLNPs was tested against Gram-positive (S. aureus) and Gram-negative (P. aeruginosa and E. coli) utilising the standard broth microdilution method [42]. Initially, the bacteria cultures were grown in MHB medium overnight (37 °C and 230 rpm). Subsequently, these cultures were diluted by using MHB to achieve the optical density at 600 nm (OD_600_) as 0.01 (corresponding to the concentration of bacteria of ∼10^5^-10^6^ CFU/mL (colony-forming units per millilitre)). Further, 50 μL of bacterial suspension (OD_600_ = 0.01) was added to 50 μL dispersed AgPLNP solutions at different concentrations (2.5, 1.25, 0.625, 0.313, 0.156, and 0.078 mg/mL) in 96-well microplates. After mixing, the initial OD_600_ was measured using an Infinite M200 Tecan microplate reader, Austria. The samples were then incubated for 24 h at 37 °C and 230 rpm in a dynamic incubator. Following incubation, bacterial growth in the presence of NPs was assessed by measuring the OD_600_ again. A bacterial culture without NPs served as the control, showing no growth inhibition. The degree of growth inhibition in the treated samples was calculated based on the OD_600_ measurements.

$$Bacterial\;growth\;inhibition\;\left(\%\right)=100\;-\left(\frac{{OD}_{t=\;24}-{\;OD}_{t\;=\;0}}{{OD}_{growth\;control}-{\;OD}_{t\;=\;0}}\;x\;100\right)$$

where, OD_t=0_ represents the absorbance measured immediately after mixing the NPs with bacteria; OD_t=24_ represents the absorbance measured after 24 h of incubation, and OD_growth control_ is the absorbance of bacteria without any treatment.

The lowest amount of AgPLNPs required to impede the growth of bacteria by 99.9 % after 24 h is defined as the minimal inhibitory concentration (MIC). The minimum bactericidal concentration (MBC) was determined by plating 10 μL of bacterial suspensions on selective agar, followed by incubation at 37 °C in a static incubator for 24 h. After incubation, bacterial colonies were counted to assess bacterial viability.

#### 2.3.4 Determination of bacterial metabolic activity

The effect of AgPLNPs on bacteria's metabolic activity was evaluated using the resazurin assay, in which the bacterial suspensions (OD_600_ = 0.01) were initially incubated with the AgPLNPs for 24 h at 37 °C and 230 rpm in a dynamic incubator, followed by addition of 10 μL of resazurin solution (100 μg/mL) to each well and incubation for 4 h. After 4 h of incubation, the fluorescence intensity was measured at 520/590 nm excitation/emission wavelengths to assess the cells' viability using the following equation.

$$Metabolic\;activity\;\left(\%\right)=\left(\frac{{FI}_{sample}-{FI}_{blank}\;}{{FI}_{control}-{FI}_{blank}}\right)x\;100$$

where FI_sample_ represents the fluorescence intensity measured after 24 h of incubation, FI_blank_ represents the fluorescence intensity of the blank samples without cells, and FI_control_ is the fluorescence intensity of the control samples without NPs.

#### 2.3.5 Antimicrobial resistance of AgPLNPs

The AMR development potential of AgPLNPs against pathogenic bacteria, specifically *S. aureus* and *E. coli,* was also evaluated. The experiment began by determining the minimum inhibitory concentration (MIC) for AgPLNPs, ciprofloxacin (for *S. aureus*), and ampicillin (for *E. coli*) on day 1. A daily process was then implemented to assess potential resistance development. Each day, bacteria surviving at the highest concentration of NPs or antibiotics from the previous day's test were selected. These survivors were diluted (1: 50) and used as the starting population for the next day's MIC assay. This cycle continued for 30 consecutive days, with MIC values measured and recorded throughout the experiment. This approach allowed for tracking changes in bacterial susceptibility to the NPs and conventional antibiotics over time, providing insights into the potential for resistance development.

#### 2.3.6 Enzymatic activity of laccase

Laccase activity of both Sigma-Aldrich and industrial laccase was assessed using a microplate reader (Infinite M200, Tecan, Austria) by measuring the absorbance at 436 nm of a 5 mM ABTS solution in 100 mM sodium acetate buffer (pH 5), oxidised by laccase.

### 2.4 Batch mode sono-enzymatic functionalisation of cotton fabrics with AgPLNPs

For coating AgPLNPs on the cotton fabrics, the Ti-horn of the ultrasonic transducer was immersed approximately 1 cm deep and centrally positioned in the reaction vessel containing 200 mL aqueous solution of AgPLNPs (10 mg), GA (0.3 % w/v), 200 μL of Sigma-Aldrich laccase (448.6 ± 21.2 U/mL), and the textile sample (10 × 10 cm). The total enzyme activity in the batch system was approximately 90 U per 200 mL reaction volume (equivalent to 450 U/L). The coating process was carried out for 30 min using a Ti-horn (20 kHz, Sonics and Materials VC750, USA) with an intensity of 17.30 W/cm^2^, power density of 0.43 W/cm^3^, power output of 21.5 W, and 35 % amplitude at 50 °C. After coating, the cotton fabric was thoroughly washed using deionised water to eliminate loosely bound AgPLNPs and GA. In this study, we have compared three modifications: i) cotton fabric ultrasonicated only with AgPLNPs (M1); ii) cotton fabric ultrasonicated with AgPLNPs and 200 μL of laccase (M2); and iii) cotton fabric ultrasonicated with AgPLNPs, GA (0.3 % w/v), and 200 μL of laccase (M3).

### 2.5 Characterisation of the coated fabrics

#### 2.5.1 Chemical properties of the coated fabrics

Attenuated total reflectance-Fourier transform infrared (ATR-FTIR) spectra of different coated and uncoated cotton fabric samples (dimensions: 0.5 x 0.5 cm) were acquired over 650–4000 cm^−1^ using a PerkinElmer Spectrum 100 FTIR spectrometer, USA. Each spectrum was recorded with 64 scans at a 4 cm^−1^ resolution.

#### 2.5.2 Surface morphology of the sono-enzymatically coated textiles

The surface morphology of the coated textiles was observed under a scanning electron microscope (FE-SEM Zeiss Merlin microscope, Carl Zeiss Microscopy GmbH (Jena, Germany)).

#### 2.5.3 Phenolic content of the coatings

The availability of free phenolic groups on the surface of the coated textiles was evaluated by calculating the total phenolic content using the Folin-Ciocalteu assay [14]. A coated fabric (10 mg) was incubated with 20 % w/v Na_2_CO_3_ solution (120 μL), 0.2 N Folin-Ciocalteu reagent (70 μL) and deionised water (1.3 mL). The mixture was then incubated in the dark at room temperature for 2 h before measuring the absorbance at 765 nm using a microplate reader. The results are presented as mean values ± standard deviations (n = 3).

#### 2.5.4 Surface wettability of the coated fabrics

The surface wettability was evaluated using the sessile drop methodology on a Krüss DSA 25 (Krüss, Germany) employing Krüss Advanced (v1.13.0.21301) software. The tangential approach was used to estimate the water contact angle (WCA). The results are presented as mean values ± standard deviations (n = 3).

#### 2.5.5 Silver release from the coating

The silver release from the coated textiles was evaluated using the Model 7800 ICP-MS system from Agilent with a limit of detection (LoD) of 6.34 ppb and limit of quantification (LoQ) of 21.14 ppb for ^107^Ag. Cotton fabric samples (1 × 1 cm) were incubated in 1 mL of PBS (37 °C and 100 rpm), and the solution was exchanged after 1, 3, 5, 10, 24, 48, 72, 96, 120 and 168 h. The collected solutions were treated with 1 mL of 20 % HNO_3_ solution and diluted to obtain a final concentration of HNO_3_ of 2 % before the ICP-MS analysis. The results are presented as mean values ± standard deviations (n = 3).

#### 2.5.6 Antibacterial activity of the coated fabrics

The antibacterial activity of coated cotton samples (1 x 1 cm) was assessed using the conventional shake-flask approach designed for testing coated fabrics. This process evaluates the antibacterial activity by measuring the reduction in bacterial colonies, expressed as the average CFU/mL. The bacterial culture was prepared for antibacterial activity by incubating a single bacterial colony in MHB overnight at 37 °C with shaking at 230 rpm. The resultant culture was diluted in PBS to obtain the suspension with OD_600_ as 0.28 ± 0.01, corresponding to a bacterial concentration of ∼1.5–3.0 × 10^8^ CFU/mL. The bacterial sample was again diluted to obtain the final concentration of ∼1.5–3.0 × 10^5^ CFU/mL. Next, cotton fabrics (1 x 1 cm) were exposed to 1 mL bacterial suspension (concentration ∼1.5–3.0 × 10^5^ CFU/mL) for 4 h at 37 °C with shaking at 230 rpm. Following incubation, the suspensions were serially diluted using PBS, plated on plate-count agar, and incubated at 37 °C for 24 h. Further, the antibacterial activity was determined by comparing the average number of surviving bacteria before (A) and after (B) incubation with the treated cotton textile samples. The results are presented as mean values ± standard deviations (n = 3).

#### 2.5.7 Durability of the coating

The stability of the coating was evaluated by subjecting the coated fabrics (sample dimensions: 5 x 5 cm) to up to 60 washing cycles following hospital thermal disinfection protocols. The washing protocol was developed in line with international guidelines for healthcare textiles, which recommend thermal disinfection rather than high-pressure sterilisation [43,44]. Washing was performed in a laboratory washing machine (Ahiba Nuance, Datacolor, USA) at 75 °C with a non-ionic surfactant (Cotemol NI) at 0.1 g/L concentration. Each washing cycle lasted 15 min with agitation at 30 rpm. This temperature corresponds to the standard thermal disinfection protocol for reusable medical textiles (70–80 °C), which differs from the high-pressure sterilisation process applied to surgical instruments (121 °C, 15 psi). After every five cycles, the antibacterial activity of the fabrics was evaluated, providing insights into the long-term efficacy of the coating upon use. The results are presented as mean values ± standard deviations (n = 3).

#### 2.5.8 Kinetics of bacterial eradication

The minimal time required for the coated fabric to achieve bacterial elimination was evaluated through a time-dependent bacterial reduction experiment. Bacterial cultures, grown overnight in MHB, were used to prepare suspensions with a final concentration of approximately 1.5–3.0 x 10^5^ CFU/mL in sterile PBS. A 1 mL aliquot of this bacterial suspension was added to a textile sample (1 x 1 cm) and incubated in a shaking incubator at 37 °C. The bacterial reduction at various time points was quantified using the drop plate method. The results are presented as mean values ± standard deviations (n = 3).

#### 2.5.9 Biocompatibility of the coated fabrics

The biocompatibility of the coated fabric was evaluated against mammalian cell lines (keratinocytes and fibroblasts). After seeding the cells, they were exposed to the textile samples for 72 h. The cell viability was assessed with the Alamar Blue, as previously described [38]. The results are shown as mean values ± standard deviations (n = 3). Furthermore, the distribution of human cells was evaluated using the LIVE/DEAD kit test. The cells were treated in the dark for 15 min with a stain mixture in a 1: 4 ratio of ethidium to calcein homodimer in PBS. The cells were washed with PBS to remove unbound stains and analysed using a fluorescence microscope.

### 2.6 Upscaling the sono-enzymatic coating process

A R2R sonochemical coating pilot was employed to upscale the treatment of cotton fabrics (5 × 0.5 m) with AgPLNPs, and to assess the industrial feasibility of the M3 modification. This represents a substantial scale-up from the 0.01 m^2^ laboratory samples (10 × 10 cm) to 2.5 m^2^ pilot-scale processing in a single continuous operation. The chosen fabric dimensions ensure a uniform sono-enzymatic coating across the fabric surface. The sonochemical treatment was carried out in a 12 L tank filled with preheated to 50 °C deionised water. Then, 2 g of AgPLNPs, 12 g of GA, and 120 mL of industrial laccase (43.6 ± 3.4 U/mL) were introduced. The total enzyme activity in the R2R pilot system was approximately 436 U per 12 L bath volume (equivalent to 36 U/L). The coating procedure was conducted at three speeds (0.6, 0.2, and 0.1 m/min) depending on the time required to coat the 5 m-long fabric (Table S3).

The antibacterial activity was determined as described in Section 4.5.6. Fabric samples were collected at one-metre intervals to assess the uniformity of the coating. Then, the durability of the pilot-scale coated fabrics was evaluated as described in Section 4.5.7.

### 2.7 Statistical analysis

All data are expressed as mean ± standard deviation (SD). Statistical analyses were conducted using GraphPad Prism software (version 5.04). For multiple group comparisons, one-way ANOVA was performed followed by Tukey’s post hoc test. In cases involving two groups, an unpaired two-tailed Student’s *t*-test was used. A p-value less than 0.05 was considered statistically significant, with p < 0.05 (*), p < 0.01 (**), and p < 0.001 (***) indicating increasing levels of significance.

## 3 Results and discussion

### 3.1 Synthesis and characterisation of AgPLNPs

Hybrid metal/bio-based AgPLNPs were synthesised through a safe-by-design methodology, where phenolated lignin acted as a reducing and stabilising agent for Ag^+^. The application of US further promoted the reduction of Ag^+^, ensuring uniform NP formation, improved reaction kinetics, and better NP dispersion. As a natural polymer rich in phenolic groups, lignin also imparts antioxidant activity, biocompatibility, and biodegradability to the AgPLNPs [45].

**Fig. 1:**
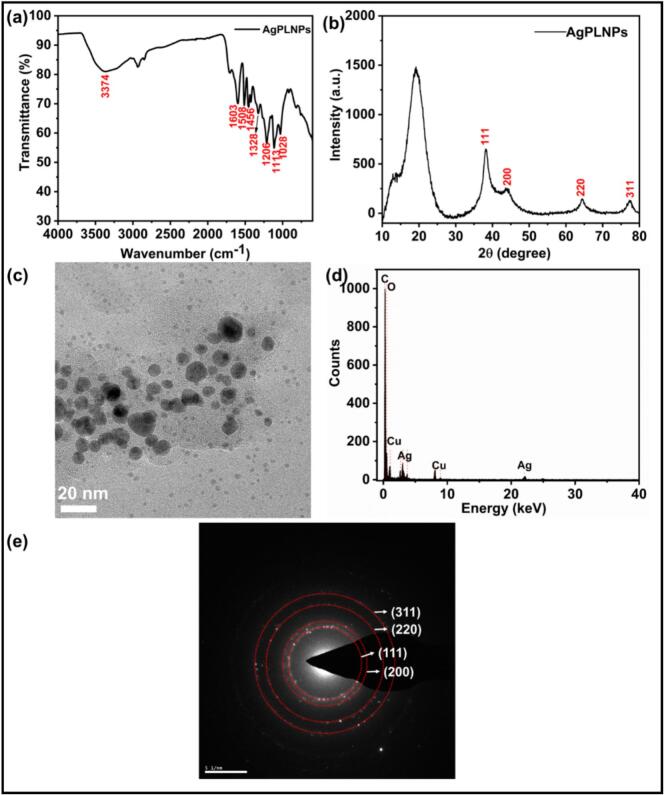
(a) FTIR spectrum, (b) XRD spectrum, (c) TEM image (scale bar = 20 nm), (d) EDX spectrum, and (e) SAED pattern of AgPLNPs.

FTIR analysis further revealed the presence of hydroxyl groups, aromatic rings, and changes in C-O and C-H bending vibrations, indicating interactions between phenolated lignin and Ag ions (Fig. 1 (a)). The broad absorption band around 3374 cm^−1^ was attributed to the –OH groups from lignin. The peaks at 1603 and 1508 cm^−1^ were ascribed to aromatic C=C stretching, while the peak at 1456 cm^−1^ corresponds to the C-H bending vibrations of methyl and methylene groups of lignin. The C-O peaks are observed at 1328, 1206, 1113, and 1028 cm^−1^, indicating the presence of alcohol and ether groups that could coordinate with Ag ions and form stable AgPLNPs [38].

XRD analysis determined the crystalline structure and phase composition of AgPLNPs (Fig. 1 (b)). The diffraction peaks corresponding to different Miller indices, specifically (111), (200), (220), and (311), appearing at 38.16°, 43.93°, 64.35°, and 77.52°, respectively, indicated that AgPLNPs possess a face-centred cubic (FCC) crystal structure, characteristic of silver. These peaks confirmed the well-ordered crystalline nature of the hybrid metal NPs. The broad peak around 19° could be associated with amorphous background material, i.e., phenolated lignin. The TEM image (Fig. 1 (c)) revealed that the AgPLNPs have a spherical form with an average diameter of 8.2 ± 2.0 nm (n = 30 particles measured). The EDX spectrum (Fig. 1 (d)) confirmed the elemental composition of the NPs, showing the presence of silver (from AgPLNPs), copper (from the supporting grid) and oxygen. The selected area electron diffraction (SAED) pattern (Fig. 1 (e)) displayed diffraction rings indexed to the (111), (200), (220), and (311) planes of Ag, confirming the NPs’ crystallinity, further supported by the XRD data.

The zeta potential of –29 mV indicated good colloidal stability of the AgPLNPs, attributed to the negative surface charge of phenolated lignin, which prevents agglomeration. Dynamic light scattering (DLS) analysis in ultrapure water revealed a hydrodynamic diameter of 192 ± 2 nm with a PDI of 0.140 ± 0.002, confirming a narrow size distribution and stable dispersion in aqueous medium (Supplementary Fig. 1). The markedly larger hydrodynamic diameter compared to the TEM size (8.2 ± 2.0 nm) is characteristic of nanoparticles with organic coatings and reflects the hydrated particle size, including the solvation shell and loosely bound lignin molecules in solution. The phenolic content of AgPLNPs was determined as 162 ± 6 mg GA equivalents per g of AgPLNPs. ICP-MS analysis showed that the dried AgPLNPs contained 12.6 ± 0.6 wt% silver, corresponding to a reduction yield of 47 % from the initial AgNO_3_. This indicates that roughly half of the silver ions were reduced and incorporated into the nanoparticles during the sonochemical process, while 87.4 wt% remained in the phenolated lignin shell and associated organic components. Such moderate reduction yields are typical of green synthesis approaches employing biological reducing agents, where the reducing capacity of phenolated lignin and the ultrasonic conditions jointly determine the extent of silver reduction. The unreduced silver was removed during the centrifugation and washing steps (Section 2.2).

### 3.2 Antibacterial activity and resistance development of AgPLNPs

The MIC of AgPLNPs was evaluated against the Gram-positive *S. aureus* and the Gram-negative *P. aeruginosa* and *E. coli* bacteria, commonly found on hospital textiles such as bedsheets and gowns [46]. The determined MIC values for AgPLNPs were 0.63 mg/mL for *S. aureus*, 0.31 mg/mL for *P. aeruginosa*, and 0.63 mg/mL for *E. coli* (Supplementary Fig. 2 (a)). The bacterial viability was evaluated using the resazurin assay. Resazurin is a non-toxic blue dye that serves as an indicator of metabolic function. When resazurin is added to bacteria, viable and metabolically active cells reduce it to resorufin, a pink, fluorescent compound. After exposure to AgPLNPs, a significant reduction in the conversion rate of resazurin was observed, indicating that the bacterial cells were damaged, and their metabolic activity was suppressed compared to the control samples without NPs (Supplementary Fig. 2 (b)). These results confirmed the antibacterial activity of AgPLNPs against the tested bacterial strains. The observed MIC variations between bacterial strains reflect differences in cellular architecture and strain-specific resistance mechanisms. *P. aeruginosa* demonstrated the highest sensitivity (lowest MIC), while *S. aureus* and *E. coli* showed similar susceptibility levels. The observed MIC variations between bacterial strains reflect differences in cell wall architecture and inherent resistance mechanisms. Interestingly, Gram-negative bacteria (*P. aeruginosa*) showed lower MIC values compared to Gram-positive *S. aureus*, which contrasts with the expectation that Gram-positive bacteria are more susceptible to antimicrobial agents. This can be attributed to the specific properties of AgPLNPs and their interaction mechanisms, where the phenolic lignin shell of the NPs may facilitate their penetration through the outer membrane of Gram-negative bacteria, leading to more efficient silver delivery and subsequent cellular damage.

Although AgNPs demonstrate potent antimicrobial activity, their use has been associated with developing antimicrobial resistance (AMR) in bacteria [47]. Studies have demonstrated that exposure to sub-lethal concentrations of AgNPs could induce adaptive resistance mechanisms, comprising changes in cell membrane permeability, overexpression of efflux pumps, and biofilm enhancement, which jointly reduce the efficiency of the silver NPs. Efflux pumps expel Ag^+^ from the bacterial cells, whereas the biofilm formation helps to shield the bacteria from the antimicrobial agents. AMR is a natural phenomenon wherein bacteria acquire the capacity to endure the effects of a particular antimicrobial agent following extended exposure [48]. To directly compare resistance development potential, we conducted in parallel 30-day sequential exposure studies with AgPLNPs and conventional antibiotics under identical conditions. After 30 days of sequential exposure of *S. aureus* and *E. coli* to sub-inhibitory concentrations of the conventional antibiotics ciprofloxacin and ampicillin, the surviving cells developed resistance, as reflected by a 2048 and 128-fold increase in the MIC, respectively (Table 1). This rise in the MIC demonstrates the ability of bacteria to rapidly adapt to antibiotics, likely through mechanisms such as modification of the antibiotic target, expression of efflux pumps for its active efflux, or antibiotic inactivation [49]. In contrast, exposure to sub-inhibitory concentrations of AgPLNPs resulted in only 4 and 2-fold increases in the MIC for *S. aureus* and *E. coli,* respectively, indicating that resistance did not emerge. A similar trend was observed in our previous works, reinforcing the hypothesis that the multiple antibacterial mechanisms of action of silver NPs capped with natural polyphenols, such as membrane disruption and oxidative stress induction, prevent the bacteria from developing resistance [40,45].

**Table 1:** Comparison of resistance development: fold-increase in MIC values for *S. aureus* and *E. coli* after 30 days of sequential exposure to sub-inhibitory concentrations of AgPLNPs *vs.* conventional antibiotics (n = 3). Lower values indicate less resistance development.

Material	MIC Value change	
	*S. aureus*	*E. coli*
AgPLNPs	4	2
Ciprofloxacin	2048	−
Ampicillin	−	128

### 3.3 Characterisation of AgPLNPs-coated cotton fabrics

An integrated sono-enzymatic coating process was employed to impart antimicrobial function to the cotton fabrics. In this US-assisted coating procedure, AgPLNPs were sonochemically embedded into an enzymatically polymerised GA matrix and deposited onto the fabric surface. The laccase catalyses the oxidation of GA and the phenolic groups of AgPLNPs’ shell, producing a crosslinked bioadhesive that adheres firmly to the fabrics. This study compares three modifications: cotton fabric ultrasonicated only with AgPLNPs (M1); cotton fabric ultrasonicated with AgPLNPs and laccase (M2); and cotton fabric ultrasonicated with AgPLNPs, GA, and laccase (M3).

**Fig. 2:**
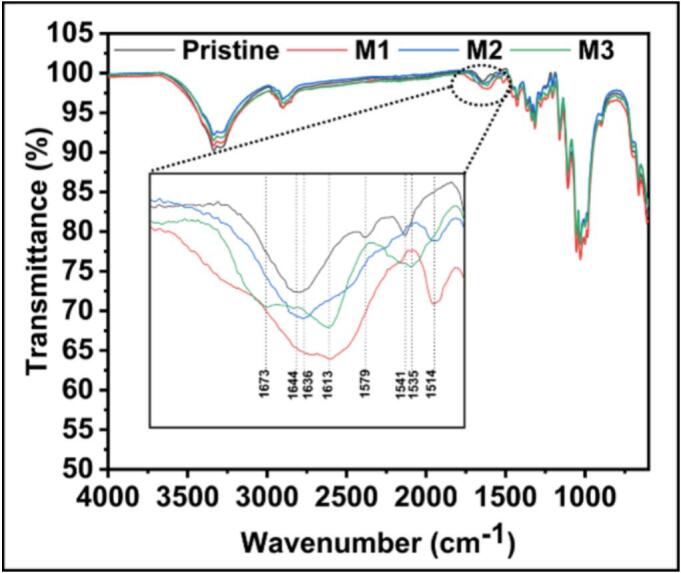
FTIR spectra of the coatings performed on cotton fabrics: pristine fabric, fabric sonicated only with AgPLNPs (M1), fabric sonicated with AgPLNPs and laccase (M2), and fabric sonicated with AgPLNPs, GA, and laccase (M3).

The coated textile samples were characterised using FTIR. In the pristine sample (Fig. 2), the peak at 1644 cm^−1^ corresponded to the O-H bending vibrations associated with the hydroxyl groups in cotton cellulose. After ultrasonication with AgPLNPs in M1, the peaks at 1613 cm^−1^ and 1514 cm^−1^ indicated the presence of C=C and C–O groups from lignin aromatic rings in AgPLNPs. In modification M3, the peak at 1673 cm^−1^ can be associated with C=O stretching of the carboxylic group of GA. The interaction of GA with AgPLNPs might have shifted the typical carboxyl peak towards the lower wavenumber. The C=C stretching vibrations of the aromatic rings from lignin and GA phenolic groups are also visible at 1613 and 1535 cm^−1^. The bioadhesive formation mechanism involves laccase-catalysed oxidation of GA phenolic groups to form reactive quinones and phenoxy radicals, which subsequently undergo coupling reactions with both the phenolic shell of AgPLNPs and other GA molecules. This dual crosslinking mechanism (GA-AgPLNP and GA-GA coupling) creates a three-dimensional covalently crosslinked network where AgPLNPs are chemically incorporated. The oxidised phenolic species also interact with cotton cellulose hydroxyl groups through hydrogen bonding and potential covalent linkages, ensuring strong adhesion to the textile substrate. The spectral changes observed in FTIR analysis provide direct evidence for chemical interactions between coating components and cotton substrate. The appearance of new peaks and shifts in existing bands confirms successful surface modification rather than simple physical deposition, supporting the proposed covalent crosslinking mechanism involving multiple molecular interactions within the bioadhesive matrix.

**Fig. 3:**
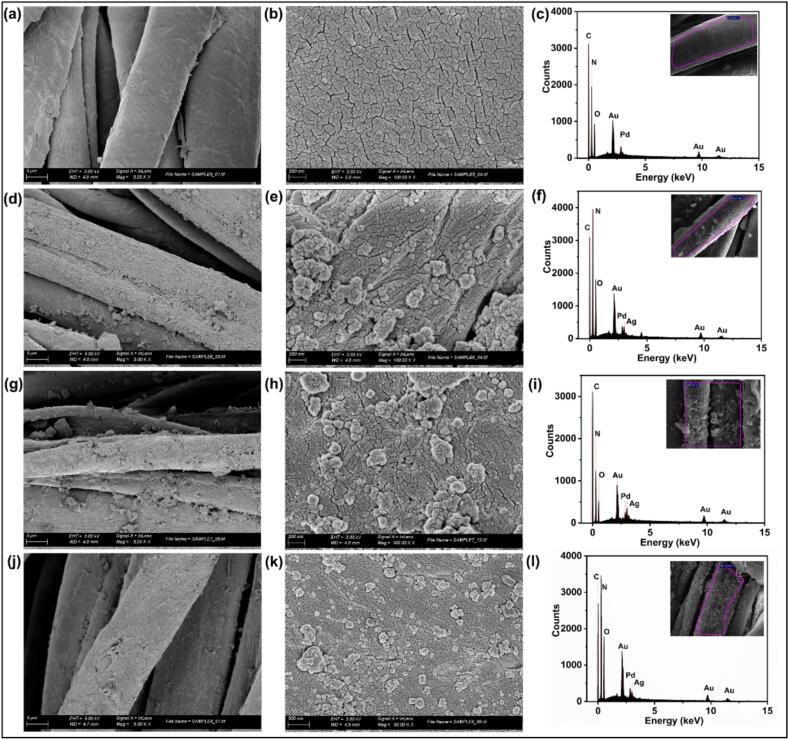
SEM images and EDX spectra of all the modifications performed on cotton fabrics: pristine cotton fabric (a-c), M1 (d-f), M2 (g-i), and M3 (j-l).

SEM images and EDX spectra revealed the modification of the surface characteristics of cotton fabrics as a function of the coatings. The pristine cotton fabric (Fig. 3a–c) exhibited a relatively smooth surface, and the EDX spectrum confirmed the presence of carbon (C) and oxygen (O) as predominant elements, consistent with the expected elemental composition of cellulose-based materials. Modification with AgPLNPs (M1) resulted in SEM images (Fig. 3 (d, e)) showing NP deposition on the surface, as confirmed by the EDX spectrum (Fig. 3 (f)), which detected Ag. In M2, when AgPLNPs were applied with laccase, NP aggregation and uneven surface coverage were observed (Fig. 3 (g, h)). The utmost significant alteration was noted in M3 (Fig. 3 (j, k)), where more uniform NP deposition was achieved on the textile surface. In these treatments, the presence of Ag on the textiles was confirmed by the EDX spectra (Fig. 3 (i, l)). The Au and Pd peaks in the EDX spectra resulted from the metallisation process, in which a thin coating of Au and Pd is sprayed onto the samples to boost surface conductivity and improve the SEM image quality. These peaks are not associated with the composition of the original textile samples.

ICP measurements further quantified the amount of AgPLNPs in the coating by evaluating the release of Ag^+^ as a determinant for the durability of the antibacterial efficacy of the fabrics (Supplementary Fig. 3 (a)). M3 exhibited the highest amount of silver compared to M1 and M2 before washing, indicating that the enzymatic crosslinking of GA and AgPLNPs significantly enhanced the latter's attachment to the cotton fabric. In contrast, the lowest Ag content was determined in M1, where the AgPLNPs were deposited onto the textile in a “throwing stone” mode without any adhesive, providing washing fastness of the coating [8].

When subjected to hospital laundry cycles (15, 30, and 60 cycles), a significant decrease in the silver content was observed across all coatings, including M3, probably due to the removal of the loosely bound AgPLNPs. Nevertheless, M3 retained the highest silver content after 60 washing cycles, demonstrating superior durability and highlighting the role of the enzymatic polymerisation and crosslinking of GA and AgPLNPs as structural components providing the coating stability. The exceptional durability of our AgPLNP-coated textiles compares favourably with recent antimicrobial textile developments [34,50]. Although several studies have reported strong initial antimicrobial activity, sustaining this performance after repeated washing remains a major challenge [35,51]. The sono-enzymatic crosslinking mechanism employed here offers superior wash-fastness compared to physical adsorption or weak chemical bonding methods commonly described in the literature [52]. This advantage is evident in our results, where M1 (physical deposition without enzymatic crosslinking) showed the most significant silver loss, highlighting is poor wash resistance in the absence of the bio-based adhesive.

M3 also exhibited the highest phenolic content (∼0.317 ± 0.007 μmol GAE/mg) among all coated textiles (Supplementary Fig. 3 (b)). The initially high phenolic content of the coating was primarily attributed to the presence of GA, whereas the phenols from the NPs in the other samples were not detected due to the method's detection limit. After several washing cycles, the phenolic content of M3 significantly decreased, presumably due to the release of loosely associated phenolics (GA and AgPLNPs), reaching a plateau after the fifth washing cycle.

### 3.4 Surface wettability

**Fig. 4:**
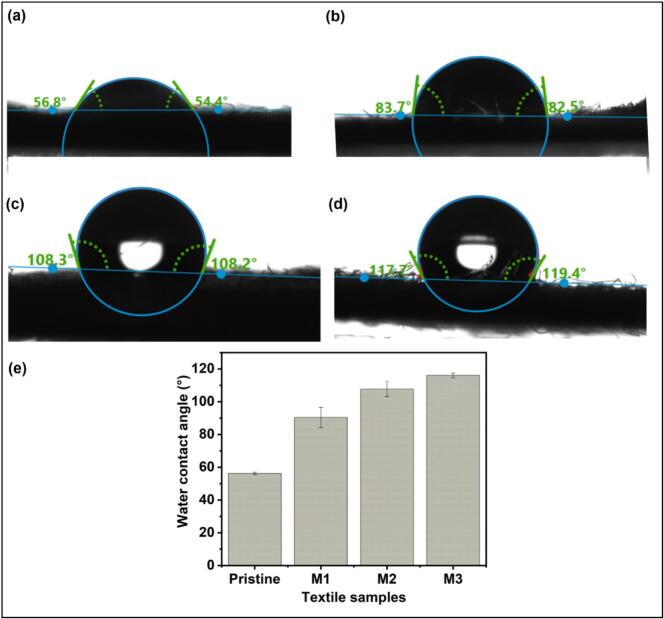
Water contact angles of (a) pristine cotton fabric, (b) M1, (c) M2, and (d) M3, and (e) measurements showing mean ± SD (n = 3).

The deposition of the NPs was confirmed through the changes in the wettability of the cotton fabrics. Surface wettability is determined by calculating the contact angle, giving insights into the hydrophobicity or hydrophilicity of the coated surfaces. An increase in the contact angle after the coating indicates that the fabric surface became more hydrophobic, leading to water repellency and reduced bacterial adhesion. The pristine fabric demonstrated moderate hydrophilicity, characteristic of untreated cotton, which inherently tends to absorb water (contact angle: 56.3 ± 0.8°) (Fig. 4 (a)) [53]. Upon deposition of AgPLNPs (M1), the contact angle of 90.4 ± 6.2° confirmed the increased hydrophobicity of the fabrics (Fig. 4 (b)). Further increase in the hydrophobicity (contact angle: 107.7 ± 4.6°) was observed after M2 (Fig. 4 (c)). Finally, in M3, the highest hydrophobicity of the fabric was detected (contact angle: 116.1 ± 1.5°), suggesting the formation of a hydrophobic surface (Fig. 4 (d)) that could potentially provide reduced bacterial adherence coupled with effective moisture protection.

This progressive increase in hydrophobicity from M1 to M3 can be attributed to the combined effects of AgPLNPs deposition and enzymatic crosslinking. In M1, AgPLNPs alone provide moderate hydrophobicity due to the phenolic lignin shell. M2 shows enhanced hydrophobicity through laccase-mediated crosslinking and partial network formation. M3 exhibits the highest hydrophobicity due to the formation of a dense, crosslinked GA-AgPLNPs network with a rougher surface topography and reduced availability of hydrophilic sites. The enzymatic crosslinking process converts the hydrophilic phenolic OH groups into less polar quinone and ether linkages, while the three-dimensional network structure traps air pockets, contributing to the observed hydrophobic behaviour.

The gentle sono-enzymatic coating conditions (50 °C, aqueous medium, mild ultrasonic intensity) and the formation of a thin surface coating suggest minimal impact on the fabric's mechanical integrity, avoiding harsh chemical treatments that typically compromise textile mechanical properties. Future work should include a comprehensive mechanical property evaluation to fully characterise the impact of sono-enzymatic coating on fabric handle and durability under mechanical stress.

### 3.5 Antibacterial performance of AgPLNPs-coated fabrics

**Fig. 5:**
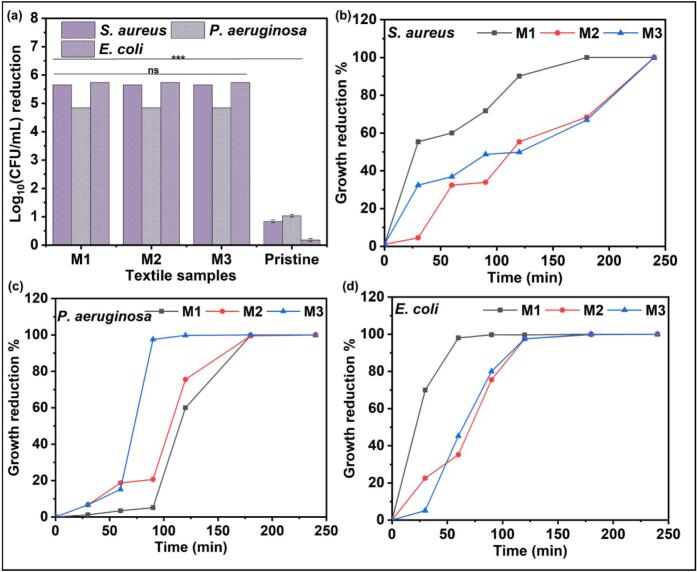
Log_10_(CFU/mL) reduction of AgPLNP-coated textiles against (a) bacterial strains after 4 h of incubation and growth reduction (%) relative to the incubation time for (b) *S. aureus*, (c) *P. aeruginosa*, and (d) *E. coli* (n = 3).

The antibacterial evaluation of the AgPLNP-coated fabrics demonstrated significant log reduction of 5.65 ± 0.0, 4.85 ± 0.0, and 5.74 ± 0.0 for *S. aureus*, *P. aeruginosa,* and *E. coli,* respectively, after 4 h of incubation (Fig. 5 (a)). When assessing the antibacterial performance of the coated fabrics as a function of time, it was observed that the killing of bacteria was slower in the case of *S. aureus* than in *P. aeruginosa* and *E. coli*. For (M3), 99.9999 % antibacterial activity (log reduction = 5.84 ± 0.0) was observed after 240 min of incubation, while *P. aeruginosa* achieved 99.75 % activity (log reduction = 2.69 ± 0.0) after 120 min, and *E. coli* achieved 99.97 % activity (log reduction = 3.54 ± 0.21) after 180 min (Fig. 5 (b-d) and Supplementary Fig. 4)*.* The strain-specific differences in bacterial elimination kinetics reflect distinct cellular defence mechanisms rather than simple structural variations. *S. aureus* demonstrated slower killing rates, possibly due to its robust cell wall structure and ability to express stress response systems. Conversely, the rapid initial killing observed for *P. aeruginosa* and *E. coli* suggests efficient disruption of essential cellular processes through multiple antimicrobial mechanisms, including membrane destabilisation and intracellular oxidative damage. This highlights the importance of accounting for the unique characteristics of bacterial strains when assessing antimicrobial activity [54].

**Fig. 6:**
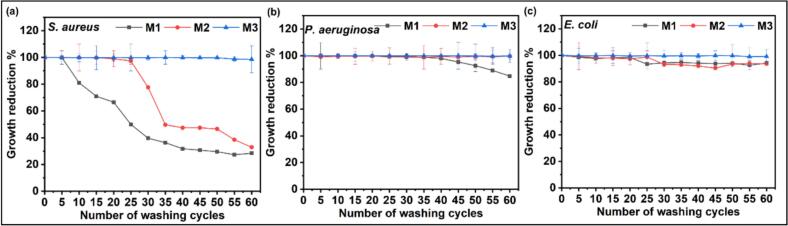
Antibacterial performance of AgPLNP coated textile samples against (a) *S. aureus,* (b) *P. aeruginosa,* and (c) *E. coli* over the washing cycles (n = 3).

The durability of the antimicrobial performance of AgPLNP-coated fabrics was assessed following exposure to multiple high-temperature hospital laundry cycles. The hospital laundry protocol follows international guidelines for healthcare textile thermal disinfection, utilising 75 °C washing temperature to ensure pathogen elimination [43,44]. In most cases, a sharp decline in log reduction was observed after the first five washing cycles due to the removal of loosely bound AgPLNPs and coating components (Supplementary Fig. 3 (a,b)), followed by a more gradual decrease over the subsequent cycles (Supplementary Fig. 5). However, the amount of AgPLNPs remaining on M3 coating was sufficient to achieve 98.62 % (log reduction = 1.86 ± 0.69), 99.81 % (log reduction = 2.73 ± 0.33), and 99.21 % (log reduction = 2.10 ± 0.22) antibacterial performance after 60 washing cycles for *S. aureus*, *P. aeruginosa,* and *E. coli*, respectively (Fig. 6). The washing resistance of AgPLNP-coated textiles was better than ZnO NPs-coated ones (previously reported by our group [14]) due to the intra- and inter-molecular crosslinking among phenolic groups of the AgPLNPs and GA forming an insoluble bioadhesive chemically embedded with the antimicrobial agent.

The release of Ag^+^ from the coated fabrics over 7 days was below the instrument's limit of detection (6.34 ppb), indicating negligible silver leaching into the PBS solution. This finding supports a contact-based antimicrobial mechanism where antibacterial efficacy results from direct interaction between surface-bound AgPLNPs and bacterial cells, rather than through released silver ions. Silver-based NPs are well-known for generating reactive oxygen species (ROS) upon contact with bacterial cells, damaging bacterial cell structures and DNA, resulting in cell death even without considerable Ag^+^ release [38]. The antibacterial efficacy of the coated fabrics may stem from a combination of direct-contact mechanisms and surface-associated activities (ROS generation, membrane disruption, protein denaturation, and electrostatic destabilisation) instead of the release of Ag^+^ into the solution. AgPLNP coating (M3) stability was also evaluated after one year of storing the textile samples under ambient conditions (average annual temperature ∼20–25 °C) in Barcelona, Spain. No significant decline in the antibacterial performance was observed, suggesting high coating durability at storage (Supplementary Fig. 6).

### 3.6 Biocompatibility evaluation

**Fig. 7:**
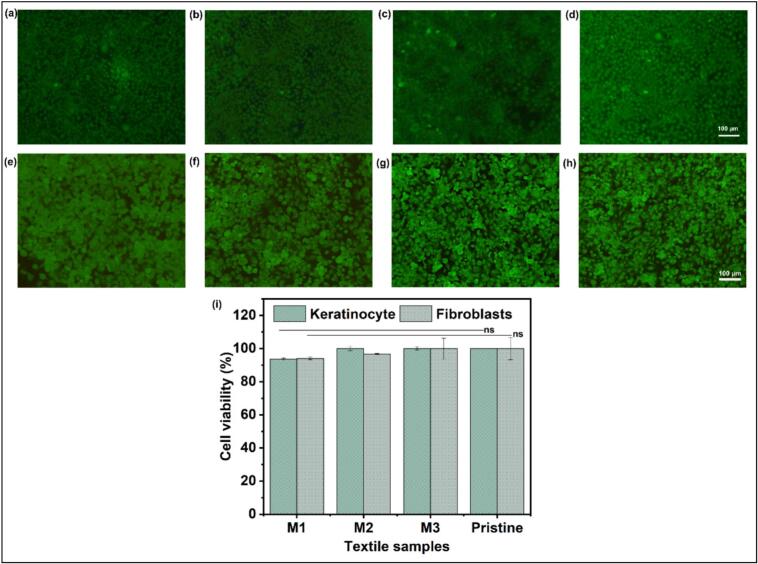
Live/Dead assay of keratinocytes (a-d) and fibroblasts (e-h) after three days of exposure to M1, M2, M3, and pristine cotton fabric, respectively. The images are captured with green and red fluorescence superimposing with each other. (i) Cell viability after three days of exposure to M1, M2, M3, and pristine textiles (n = 3).

Silver-based NPs could potentially harm humans, animals, and the environment, typically by mechanisms similar to their antibacterial action. Hence, the biocompatibility of the coated textiles was evaluated against two mammalian cell lines − keratinocytes and fibroblasts − as they are part of the human skin epidermis and dermis, respectively [55]. After three days of exposure, the cultured cells exhibited metabolic activity with no discernible differences in cell viability across coated and uncoated fabrics, validating the biocompatibility of the coatings (Fig. 7).

### 3.7 Antibacterial activity and durability of the upscaled sono-enzymatically coated fabrics

**Fig. 8:**
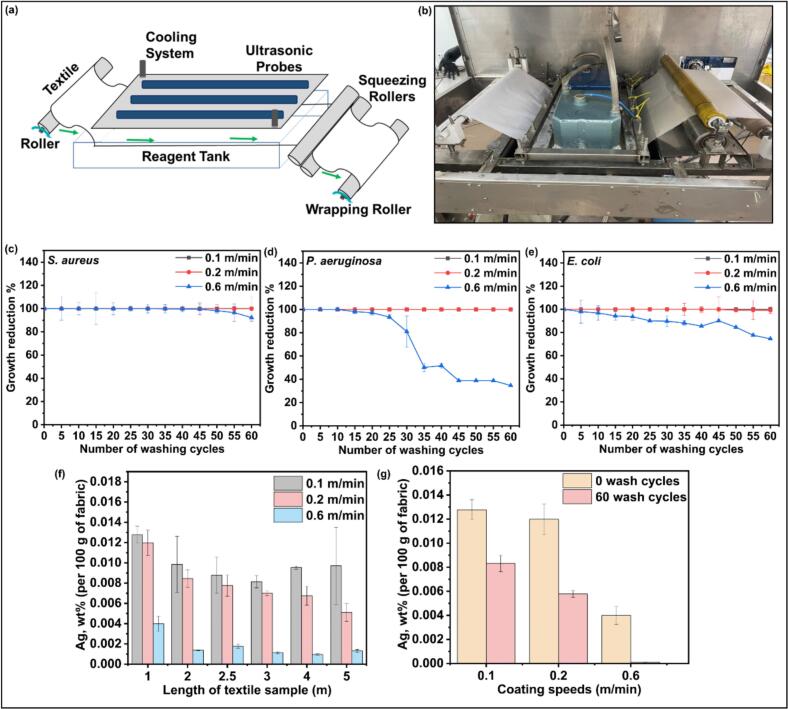
(a) Schematic illustration of R2R ultrasonic textile coating and (b) image captured during the coating process. Antibacterial performance of AgPLNP-coated fabrics (prepared at coating speeds of: 0.1, 0.2, and 0.6 m/min) against (c) *S. aureus,* (d) *P. aeruginosa,* and (e) *E. coli* over the washing cycles (n = 3). (f) Silver content of the coated fabrics (wt% per 100 g of fabric) measured at different textile sample lengths and processing speeds (n = 3). (g) Silver content before and after washing at 75 °C (n = 3).

To demonstrate industrial feasibility, the sono-enzymatic coating process was scaled up from laboratory batch set-up on 10 × 10 cm fabric samples to continuous R2R processing of 5 × 0.5 m cotton fabrics, representing a 250-fold increase in processing area (Fig. 8 (a,b)). The R2R sonochemical coating process is a scalable, continuous-mode technology suitable for large-scale production of antimicrobial textiles, avoiding the multiple handling steps of batch processing. Furthermore, the R2R process allowed for a uniform application of the functional coating on the textile substrates, an essential factor for mass production. Such a process benefits high-throughput applications where coating precision, reducing material wastage, and increasing the coating process speed are critical [27].

After the pilot coating, the antibacterial performance of the fabrics was evaluated by sampling every 1 m (from 1 to 5 m). The log reduction values of bacteria on the fabrics coated on the R2R sonochemical pilot at three different speeds, 0.6, 0.2, and 0.1 m/min, are summarised in Table 2. At a coating speed of 0.6 m/min, the fabrics exhibited a significant log reduction of 5.87 ± 0 and 6.00 ± 0 for *S. aureus* and *E. coli*, respectively, demonstrating no significant change in antibacterial performance along the fabric length. Slight fluctuations were observed for *P. aeruginosa*, showing log reductions between 4.95 ± 0 and 4.46 ± 1.6. At intermediate speed (0.2 m/min), *S. aureus* exhibited no significant fluctuations in log reduction; however, *P. aeruginosa* and *E. coli* displayed substantial variations, with log reductions varying from 3.55 ± 1.62 to 4.95 ± 0 and 4.77 ± 1.42 to 6.00 ± 0, for *P. aeruginosa* and *E. coli,* respectively. Lastly, at the slowest speed (0.1 m/min), the log reductions for all bacterial strains exhibited more fluctuations. At the slowest speed of 0.1 m/min, the more considerable log reduction variations might be attributed to the prolonged exposure of fabric sections to the ultrasonic energy and coating solution, resulting in local saturation or uneven distribution.

**Table 2:** Antibacterial activity of the upscaled sono-enzymatic coating of cotton fabric with AgPLNPs at different fabric speeds (n = 6).

Textile Sample From (meters)	Log reduction		
	*S. aureus*	*P. aeruginosa*	*E. coli*
0.6 m/min			
1	5.87 ± 0	4.95 ± 0	6.00 ± 0
2	5.87 ± 0	4.95 ± 0	6.00 ± 0
2.5	5.87 ± 0	4.95 ± 0	6.00 ± 0
3	5.37 ± 1	4.95 ± 0	6.00 ± 0
4	4.46 ± 1.6	4.95 ± 0	6.00 ± 0
5	5.87 ± 0	4.95 ± 0	6.00 ± 0
0.2 m/min			
1	5.87 ± 0	4.38 ± 1.15	5.42 ± 1.15
2	5.87 ± 0	4.95 ± 0	5.35 ± 1.30
2.5	5.87 ± 0	4.28 ± 1.34	4.77 ± 1.42
3	5.87 ± 0	3.55 ± 1.62	6.00 ± 0
4	4.37 ± 1.73	4.33 ± 1.23	4.73 ± 1.46
5	4.25 ± 1.87	3.76 ± 1.42	4.92 ± 0
0.1 m/min			
1	4.33 ± 1.78	4.45 ± 1	5.38 ± 1.23
2	5.29 ± 1.15	4.95 ± 0	4.77 ± 1.30
2.5	5.20 ± 1.34	4.30 ± 1.30	5.50 ± 1
3	4.79 ± 1.24	3.76 ± 1.38	5.42 ± 1.15
4	4.75 ± 1.30	4.28 ± 1.34	5.38 ± 1.23
5	4.68 ± 1.38	4.95 ± 0	5.00 ± 1.15

Additionally, ICP results showed that silver content was highest at the slowest speed (0.1 m/min) and lowest at the fastest speed (0.6 m/min), suggesting a direct relationship between AgPLNP deposition and coating speed (Fig. 8f). Furthermore, the silver content steadily decreased along the length of the 5-metre cotton fabric, indicating a gradual depletion of the coating material throughout the application process.

The antibacterial durability of AgPLNP-coated textiles was evaluated after 60 washing cycles. The results indicated a direct correlation between coating speed and durability, with lower coating speeds (0.1 and 0.2 m/min) yielding higher bacterial reduction after 60 wash cycles (Fig. 8 (c-e) and Supplementary Fig. 7)., The highest bacteria log reduction values, namely 5.75 ± 0.0 logs (99.9998 %) for *S. aureus*, 4.59 ± 0.0 logs (99.9974 %) for *P. aeruginosa*, and 6 ± 0.0 logs (99.9999 %) for *E. coli*, were observed after 60 washings of the fabric coated at 0.1 m/min reflecting its high coating stability. In contrast, the lowest bacterial reduction was recorded for the fabric coated at 0.6 m/min: 1.11 ± 0.15 log (92.23 %) for *S. aureus*, 0.18 log ± 0.04 (32.02 %) for *P. aeruginosa*, and 0.59 ± 0.03 log (74.07 %) for *E. coli*. The samples coated at 0.2 m/min speed exhibited intermediate antibacterial activity, with 2.94 ± 0.4 log (99.88 %) reduction for *S. aureus*, 5.14 ± 0.0 log (99.9993 %) for *P. aeruginosa*, and 2.08 ± 0.10 log (99.17 %) for *E. coli,* due to the presence of sufficient silver content even after 60 washing cycles (approximately 0.006 wt% per 100 g of fabric) as demonstrated by the ICP results (Fig. 8g). These results emphasise the important contribution of the coating speed to the long-term antibacterial activity of AgPLNP-coated textiles. After 60 washing cycles, the results demonstrated that lower coating speed (0.1 m/min) facilitates better AgPLNPs retention, leading to efficient bacteria eradication. In contrast, a higher coating speed (0.6 m/min) resulted in insufficient NP retention, reducing the antimicrobial performance of the fabric. However, time efficacy is also a key factor for large-scale production. While highly effective, the 0.1 m/min speed requires 41 min to coat the 5-metre fabric, making it less practical for industrial applications. On the other hand, 0.6 m/min, despite taking only 6 min and 15 s, resulted in poor antibacterial durability. The 0.2 m/min speed presents the best balance, requiring 25 min while achieving effective bacterial inhibition and retaining silver over multiple washings, as confirmed by ICP analysis.

### 3.8 Study limitations and future directions

Several limitations of this study should be acknowledged. First, the antimicrobial evaluation was restricted to three healthcare-associated bacterial strains; broader testing against additional clinically relevant pathogens, including antibiotic-resistant strains, fungi, and viruses, would strengthen the clinical applicability claims. Second, while the 60-cycle washing durability testing was extensive, it was conducted under controlled laboratory conditions that may not fully represent the variability of real hospital laundry operations, including different detergent chemistries, water qualities, and mechanical stress variations. Third, the biocompatibility assessment, though promising, was limited to two cell lines and 72-hour exposure periods. Extended cytotoxicity studies, sensitisation testing, and evaluation with additional relevant cell types would provide more comprehensive safety data. Fourth, although silver release was below instrumental detection limits, more sensitive analytical methods and systematic environmental impact assessment of wash effluents are needed to address potential ecological concerns and regulatory requirements.

Additionally, the industrial scalability demonstrated at pilot scale requires validation at full manufacturing scale with comprehensive economic analysis, quality control protocols, and regulatory compliance strategies. Future investigations should explore the coating's performance on diverse textile substrates, optimise process parameters for maximum efficiency, and establish standardised protocols for antimicrobial textile evaluation under clinical conditions.

## 4 Conclusion

This work reports on a durable and scalable antimicrobial nanocomposite coating for medical textiles using a single-step sono-enzymatic process. The coating approach involved enzyme-assisted crosslinking of antibacterial AgPLNPs into a bio-based GA adhesive, which was simultaneously deposited onto cotton fabrics using high-intensity US. The batch mode-coated with AgPLNPs textiles exhibited excellent durability, retaining over 95 % of their antimicrobial efficacy after 60 washing cycles. Importantly, the AgPLNPs demonstrated significantly reduced resistance development potential, with only 2–4 fold MIC increases compared to 128–2048 fold increases observed with conventional antibiotics after 30 days of exposure. The upscaled R2R coating of 5 m cotton fabric demonstrated speed-dependent antibacterial activity, with optimal uniformity achieved at 0.2 m/min. However, a gradual decrease in silver content along the fabric length indicated the need for bath replenishment strategies in production-scale systems. Fabrics coated at both 0.2 and 0.1 m/min maintained their antimicrobial efficacy after 60 washings. Nevertheless, 0.2 m/min was selected as the optimal speed, providing the best balance between antibacterial activity, NP retention, and process efficiency. This scalable and robust R2R coating method offers a promising strategy for developing practical, long-lasting, and biocompatible antimicrobial textiles for healthcare applications.

## CRediT authorship contribution statement

**Garima Rathee:** Writing – review & editing, Writing – original draft, Visualization, Validation, Methodology, Investigation, Formal analysis, Conceptualization. **Jeniffer Blair:** Resources, Methodology, Investigation, Data curation. **Antonio Puertas-Segura:** Writing – original draft, Investigation. **Kristina Ivanova:** Writing – review & editing. **Guillem Ferreres Cabanes:** Investigation, Conceptualization. **Tzanko Tzanov:** Writing – original draft, Validation, Funding acquisition, Formal analysis, Data curation, Conceptualization.

## Declaration of competing interest

The authors declare that they have no known competing financial interests or personal relationships that could have appeared to influence the work reported in this paper.
