# Supplementary material for: Scaling up the sono-enzymatic coating of cotton textiles with antimicrobial silver-phenolated lignin nanocomposites
Source: Ultrason Sonochem. 2025 Oct 9;122:107609. doi: 10.1016/j.ultsonch.2025.107609 (PMC12547008; doi:10.1016/j.ultsonch.2025.107609)
Supplement: Supplementary Data 1 [file mmc1.docx]

**Supplementary Information**

**Scaling up the sono-enzymatic coating of cotton textiles with antimicrobial silver-phenolated lignin nanocomposites**

Garima Rathee, Jeniffer Blair, Antonio Puertas-Segura, Kristina Ivanova, Guillem Ferreres Cabanes, Tzanko Tzanov*

Grup de Biotecnologia Molecular i Industrial, Departament d’Enginyeria Química, Universitat Politècnica de Catalunya (UPC-BarcelonaTech), Rambla de Sant Nebridi 22, 08222 Terrassa (Barcelona), Spain.

*Author to whom correspondence should be addressed.


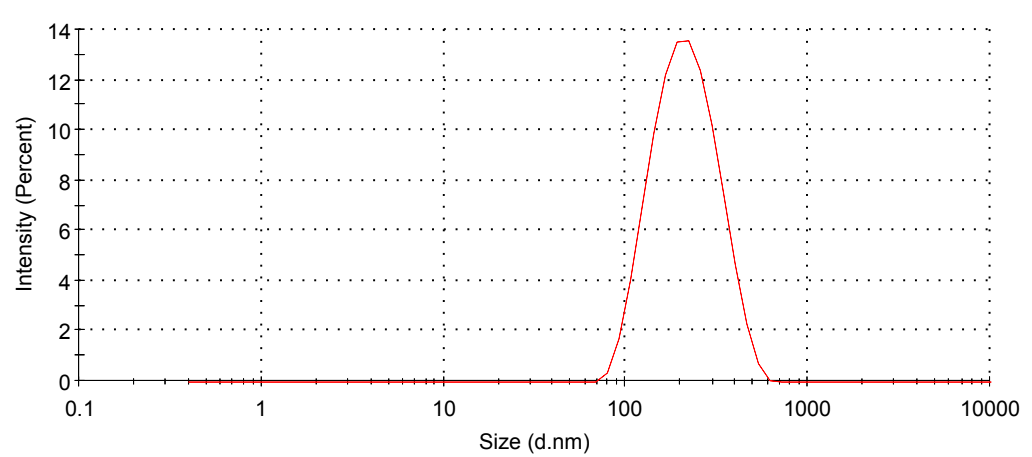
**Supplementary Fig. 1.** Hydrodynamic size distribution by intensity of AgPLNPs obtained from dynamic light scattering.


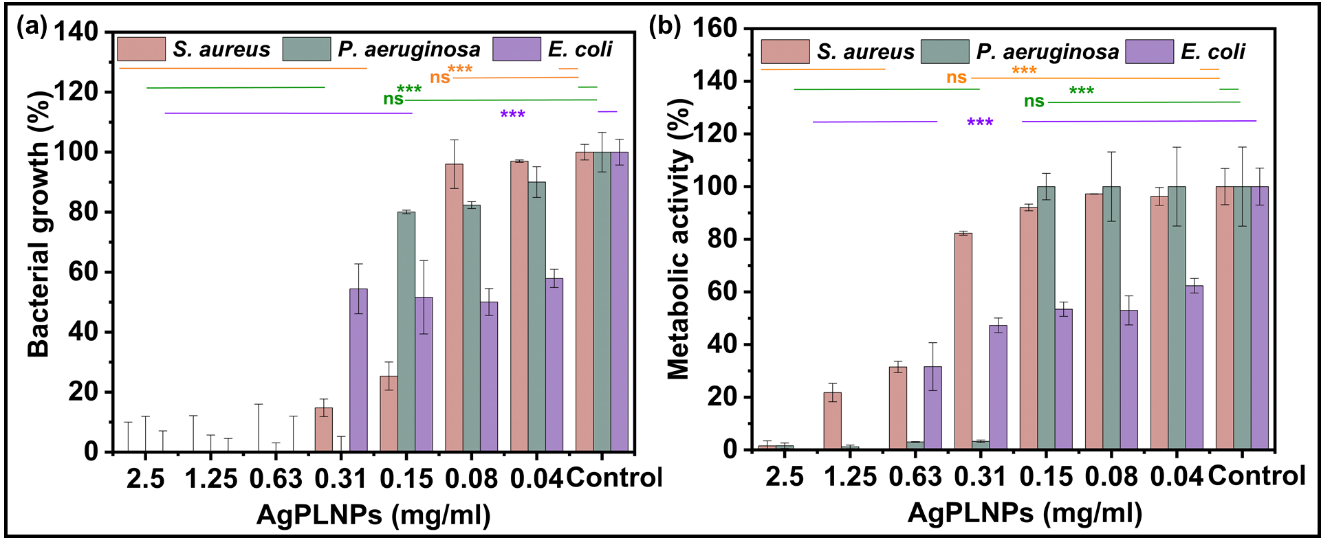


**Supplementary Fig. 2**. (a) Antibacterial efficacy of AgPLNPs against *S. aureus, P. aeruginosa* and *E. coli.* (b) Metabolic activity of bacteria subjected to different doses of AgPLNPs (n = 3).


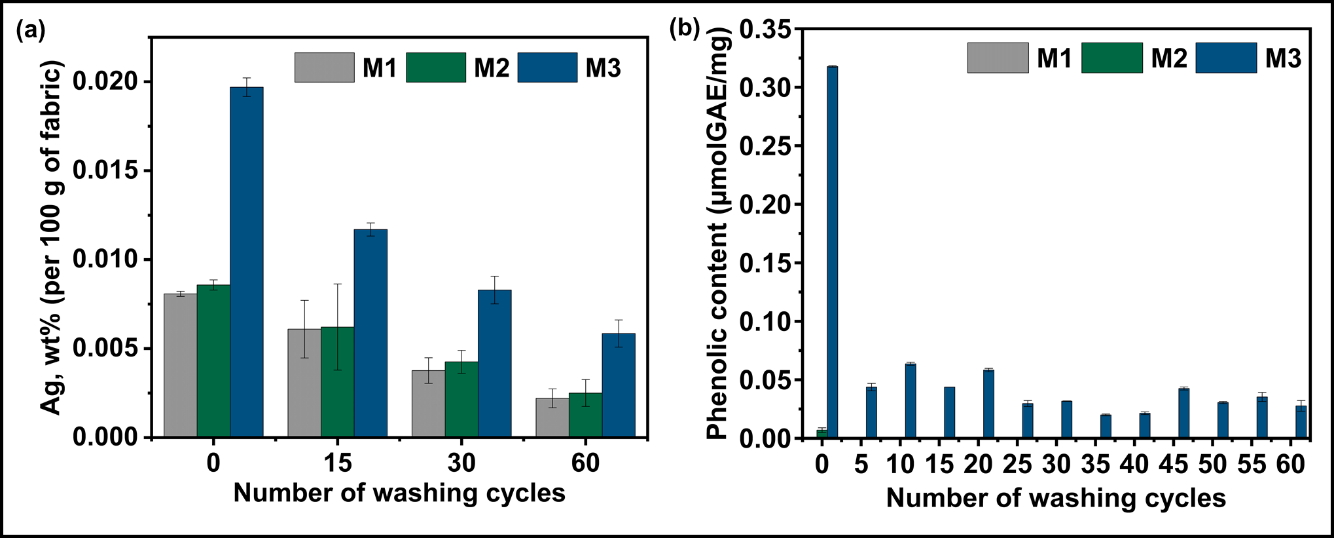


**Supplementary Fig. 3.** (a) Silver content of the coated fabrics, wt% (per 100 g of fabric) before and after washing at 75 °C (n = 3). (b) Phenolic content of the coated fabrics before and after washing at 75 °C (n = 3).


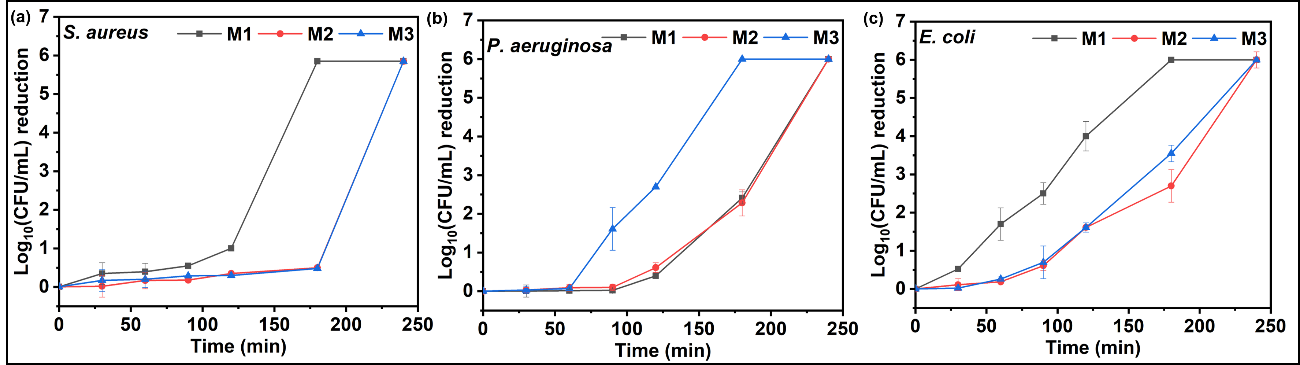


**Supplementary Fig. 4**. Antibacterial efficacy of AgPLNP coated textile samples against (a) *S. aureus,* (b) *P. aeruginosa,* and (c) *E.coli* as a function of time (n = 3).


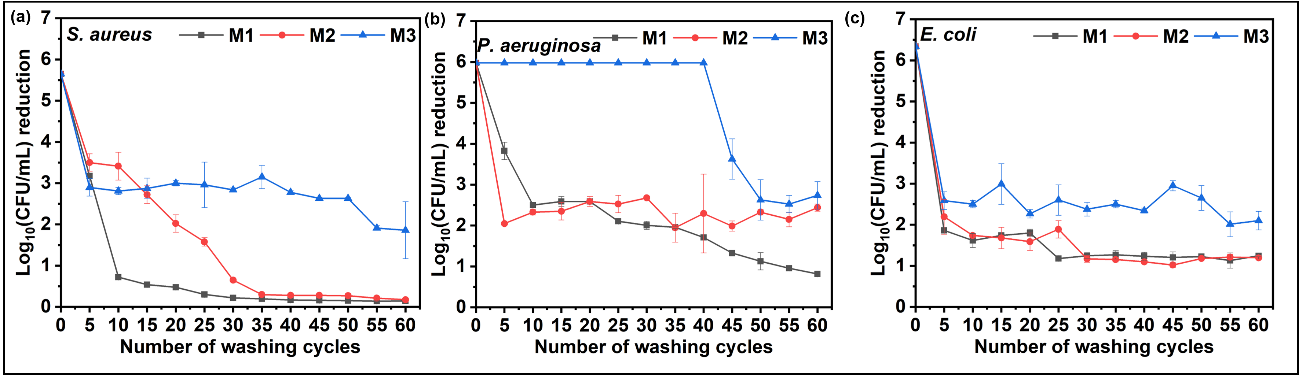


**Supplementary Fig. 5**. Antibacterial efficacy of AgPLNP-coated textile samples against (a) *S. aureus,* (b) *P. aeruginosa,* and (c) *E.coli* as a function of the number of washing cycles (n = 3).

Table 2: Laccase activity (n = 3):

| **Laccase** | **Activity** |
| --- | --- |
| Laccase from *Aspergillus* sp (Sigma-Aldrich) | 448.6 ± 21.2 U/ml |
| Industrial laccase (NewLite Base 268) | 43.6 ± 3.4 U/ml |


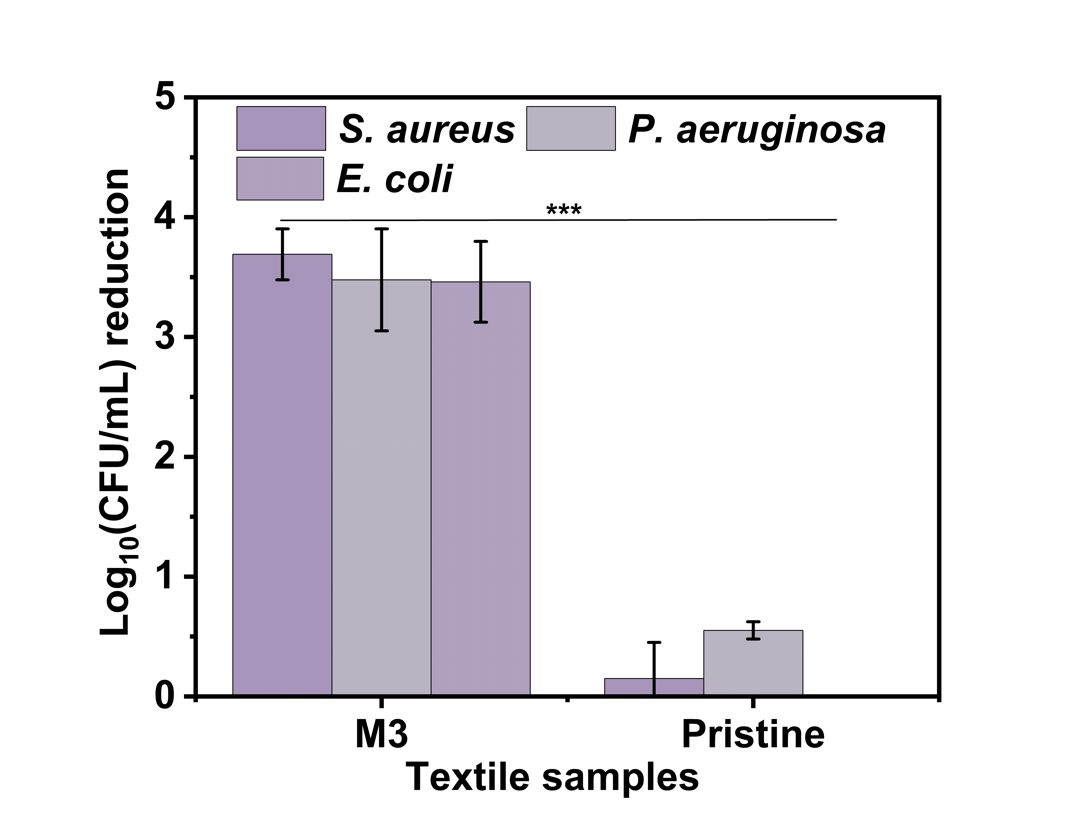


**Supplementary Fig. 6**. Bacteria log reduction by AgPLNP-coated textiles (after one year storage) upon 4 hours of incubation.

Table S3: Average time necessary to coat 5 metres of textiles at different fabric speeds in the R2R sonochemical coating pilot:

| **Speed (m/min)** | **Time for coating 5 meters of fabric** |
| --- | --- |
| 0.6 | 6 min 15 sec |
| 0.2 | 25 min |
| 0.1 | 41 min |


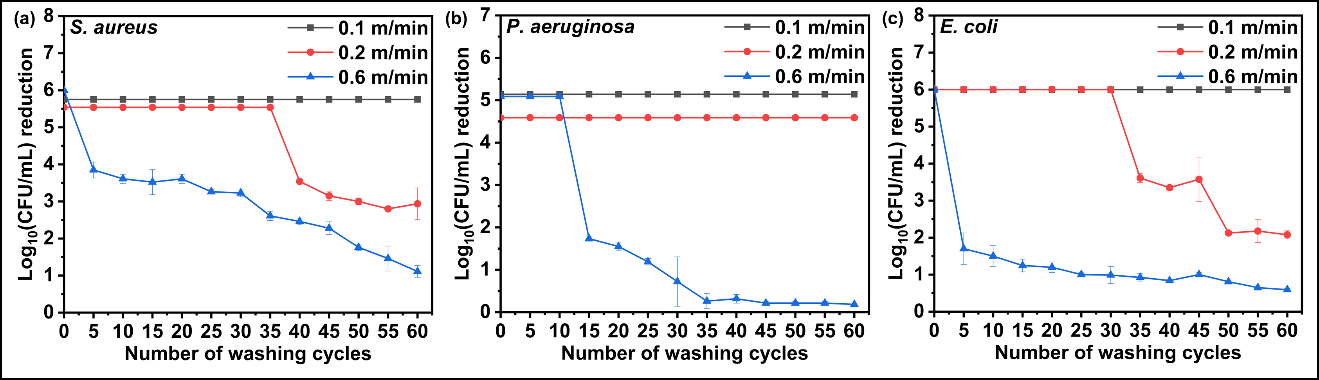


**Supplementary Fig. 7**. Antibacterial performance of upscaled AgPLNP-coated textiles against (a) *S. aureus,* (b) *P. aeruginosa,* and (c) *E.coli* depending on the number of washing cycles (n = 3).
